# Probability Distribution on Full Rooted Trees

**DOI:** 10.3390/e24030328

**Published:** 2022-02-24

**Authors:** Yuta Nakahara, Shota Saito, Akira Kamatsuka, Toshiyasu Matsushima

**Affiliations:** 1 Center for Data Science, Waseda University, Shinjuku-ku 169-8050, Tokyo, Japan; 2 Faculty of Informatics, Gunma University, Maebashi-shi 371-8510, Gunma, Japan; shota.s@gunma-u.ac.jp; 3 Department of Information Science, Shonan Institute of Technology, Fujisawa-shi 251-8511, Kanagawa, Japan; kamatsuka@info.shonan-it.ac.jp; 4 Department of Applied Mathematics, Waseda University, Shinjuku-ku 169-8555, Tokyo, Japan; toshimat@waseda.jp

**Keywords:** Bayes decision theory, Bayes statistics, recursive algorithm, rooted trees

## Abstract

The recursive and hierarchical structure of full rooted trees is applicable to statistical models in various fields, such as data compression, image processing, and machine learning. In most of these cases, the full rooted tree is not a random variable; as such, model selection to avoid overfitting is problematic. One method to solve this problem is to assume a prior distribution on the full rooted trees. This enables the optimal model selection based on Bayes decision theory. For example, by assigning a low prior probability to a complex model, the maximum a posteriori estimator prevents the selection of the complex one. Furthermore, we can average all the models weighted by their posteriors. In this paper, we propose a probability distribution on a set of full rooted trees. Its parametric representation is suitable for calculating the properties of our distribution using recursive functions, such as the mode, expectation, and posterior distribution. Although such distributions have been proposed in previous studies, they are only applicable to specific applications. Therefore, we extract their mathematically essential components and derive new generalized methods to calculate the expectation, posterior distribution, etc.

## 1. Introduction

### 1.1. Review of Literature and Motivation

Full rooted trees are utilized in various fields of study. For example, for text compression in information theory, a full rooted tree represents a set of contexts, which are strings of the most recent symbols at each time point, and it is known as a context tree [1]. In image processing, it represents a variable block-size segmentation, and it is known as quadtree block partitioning [2]. In machine learning, it represents a nonlinear function that comprises many conditional branches and is known as a decision tree [3]. In most of these studies, the rooted tree is not a random variable and serves as an index of a statistical model or function; i.e., one full rooted tree τ corresponds to one statistical model p(x;τ) or one function $f_{\tau }\left(x\right)$.

Full rooted trees’ recursive and hierarchical structures are suitable for representing complex statistical models or functional structures. For example, the expansion of the leaf nodes represents an increase in the contexts of a context tree [1], a division of a block on the image in quadtree partitioning [2], or the addition of a conditional branch in a decision tree [3]. The expressive ability and extensibility of full rooted trees render them widely applicable in various fields.

However, this hierarchical expressive capability causes a problem in tree selection, i.e., the selection of one statistical model or function. This is because the optimal tree under the criterion of the likelihood or squared loss for training data is inevitably the deepest one. This phenomenon is called overfitting in the field of machine learning. Therefore, most previous studies have applied a stopping rule for node expansion [2,3], introduced a normalization term into the objective function [4], or averaged the statistical models or the functions with some weights [1,4,5]. However, these algorithmic modifications are heuristic at times.

A theoretical way to solve this problem is to consider the full rooted tree as a random variable and assume a prior distribution on it. An appropriate prior distribution provides a unified method for selecting one full rooted tree or combining them based on Bayes decision theory (see, e.g., [6]). Although Bayes decision theory is typically applied to statistical models with unknown continuous parameters, it is also applicable to statistical models with unknown discrete random variables, such as full rooted trees (see, e.g., [7]). By assigning a high prior probability to a shallow tree and a low prior probability to a deep tree, we can avoid the complex statistical model corresponding to a deep tree.

As mentioned above, most previous studies regard a full rooted tree as a non-stochastic variable. However, a few studies adopted the above-mentioned approach. In terms of text compression, the complete Bayesian interpretation of the context tree weighting method was first investigated by the authors of [8]. Not only the theory, but also the associated algorithm have been improved during the decade they were first investigated (see, e.g., [9]). Moreover, similar results obtained from rich real data analysis have been reported recently [10,11] (note that the prior form reported in [10,11] is extremely restricted and cannot be updated as a posterior, in contrast to that reported in [8,9]). In image processing, the authors of [12] were the first to regard the quadtree as a stochastic model, and its optimal estimation was derived under the Bayesian criteria. In machine learning, the authors of [13] redefined the decision tree as a stochastic generative model and improved most tree weighting methods (e.g., [5]).

However, these studies depend on specific data or generative models. This might have been the reason that more than 25 years passed before the first study [8] pertaining to text compression was applied to image processing [12] and machine learning [13].

### 1.2. The Objective of This Study

Therefore, we separate the mathematically essential component of the discussion from the modifiable component based on specific data or the generative model. Mathematically, a tree is defined as a connected graph without cycles (see, e.g., [14]). A rooted tree is a tree that has one node known as a root node, and a full rooted tree is a rooted tree in which each inner node has the same number of child nodes. Subsequently, we can define a finite set of subtrees of a full rooted tree. This full rooted tree, which contains all the subtrees in the finite set, is denoted as a base tree herein.

A trivial method to define a probability distribution for this set is to assign occurrence probabilities to all subtrees and regard these values as parameters. In other words, we can define the categorical distribution for the finite set of subtrees of the base tree. However, this definition requires the same number of parameters as the subtrees, which increases in a doubly exponential order with the depth of the base tree.

Therefore, we propose an efficient parametric representation of the probability distribution of a set of subtrees. It is suitable for the recursive structure of full rooted trees and allows the number of parameters to be reduced. Moreover, it enables us to calculate its mode, expectation, posterior distribution, etc., using recursive functions. Therefore, it is efficient from a computational viewpoint. Furthermore, we expect these recursive functions to be effective as a subroutine of the variational Bayesian method and the Markov chain Monte Carlo method in hierarchical Bayesian modeling (see, e.g., [15]).

Strictly speaking, our distribution has already been proposed independently in source coding [8], image processing [12], and machine learning [13], as mentioned above. The substantial novelty of our study is the extraction of the essence from the previous discussion, which depends on the applicational objects, and its representation as a clear mathematical theory. This theoretically expands the potential application of probability distributions on full rooted trees. Subsequently, we derive new generalized methods to evaluate the characteristics of the probability distribution of full rooted trees, which could not be derived in previous studies pertaining to real-world applications. More precisely, only Theorems 1 and 3 and Corollary 2 has been used in previous studies. Meanwhile, the other methods expand the possibility of applying the probability distribution on full rooted trees.

### 1.3. Organization of This Paper

The remainder of this paper is organized as follows: In Section 2, we present the notation used herein. In Section 3, we define the prior for full rooted trees. In Section 4, we describe the algorithms for calculating the properties of the proposed distribution—e.g., a marginal distribution for each node, an efficient calculation of the expectation, mode, and the posterior distribution. In Section 5, we discuss the usefulness of our distribution in statistical decision theory and hierarchical Bayesian modeling. In Section 6, we propose some future work. In Section 7, we conclude the paper.

## 2. Notations Used for Full Rooted Trees

**Figure 1:**
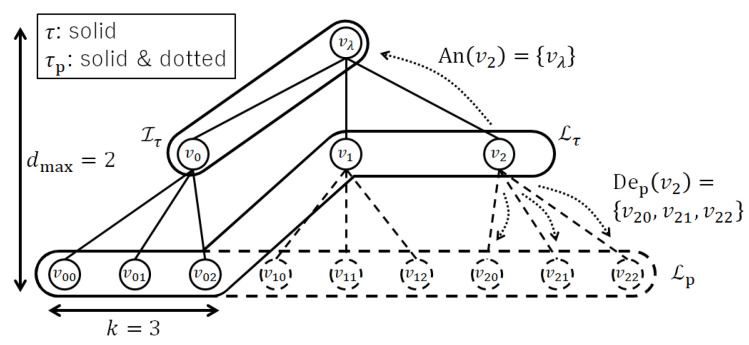
The notation for the rooted trees.

In this section, we define notation for the rooted trees. It is shown in Figure 1. Let k∈N denote the maximum number of child nodes and $d_{\max}\in N$ denote the maximum depth. Let $\tau_{p}=\left(V_{p},E_{p}\right)$ denote the perfect (“perfect” means that all inner nodes have exactly *k* children and all leaf nodes have the same depth) *k*-ary rooted tree whose depth is $d_{\max}$, and root node is $v_{\lambda }$. $V_{p}$ and $E_{p}$ denote the sets of the nodes and edges of it, respectively. Then, let $I_{p}\subset V_{p}$ and $L_{p}\subset V_{p}$ denote the set of inner nodes and the set of leaf nodes of $\tau_{p}$, respectively. For each node $v\in V_{p}$, ${\mathrm{Ch}}_{p}\left(v\right)\subset V_{p}$ denotes the set of child nodes of *v* on $\tau_{p}$. Notation about the relation between two nodes $v,v^{\prime }\in V_{p}$ is as follows. Let $v≻v^{\prime }$ denote that *v* is an ancestor node of $v^{\prime }$ ($v^{\prime }$ is a descendant node of *v*), $v⪰v^{\prime }$ denote that *v* is an ancestor node of $v^{\prime }$ or $v^{\prime }$ itself ($v^{\prime }$ is a descendant node of *v* or *v* itself), $\mathrm{An}\left(v\right):=\left\{v^{\prime }\in V_{p}|v^{\prime }≻v\right\}$, and ${\mathrm{De}}_{p}\left(v\right):=\left\{v^{\prime }\in V_{p}|v≻v^{\prime }\right\}$.

Subsequently, we consider rooted subtrees of $\tau_{p}$ in which their root nodes are the same as $v_{\lambda }$ and all inner nodes have exactly *k* children. They are called full rooted subtrees and $\tau_{p}$ is called a base tree. Let T denote the set of all full rooted subtrees of $\tau_{p}$. Let $V_{\tau }$ and $E_{\tau }$ denote the set of the nodes and the edges of τ∈T, respectively. Let $I_{\tau }\subset V_{\tau }$ and $L_{\tau }\subset V_{\tau }$ denote the set of the inner nodes and the set of leaf nodes of τ∈T, respectively.

## 3. Definition of Probability Distribution on Full Rooted Subtrees

In this section, we define a probability distribution of full rooted subtrees T. Let *T* denote the random variable on T and τ denote its realization.

**Definition** **1.** *For ${\left(\alpha_{v}\right)}_{v\in V_{p}}\in {\left[0,1\right]}^{|V_{p}|}$, we define the probability distribution p(τ) on T as below.*

(1) $$\begin{array}{c} p\left(\tau \right):=\prod_{v\in I_{\tau }}\alpha_{v}\prod_{v^{\prime }\in L_{\tau }}\left(1-\alpha_{v^{\prime }}\right), \end{array}$$

*where $\alpha_{v}=0$ for $v\in L_{p}$.*

Intuitively, $\alpha_{v}$ represents the probability that *v* has child nodes under the condition that *v* is contained in the tree (it will be proved as a theoretical fact in Remark 2). Therefore, the occurrence probability of a full rooted subtree exponentially decays as its depth increases.

**Figure 2:**
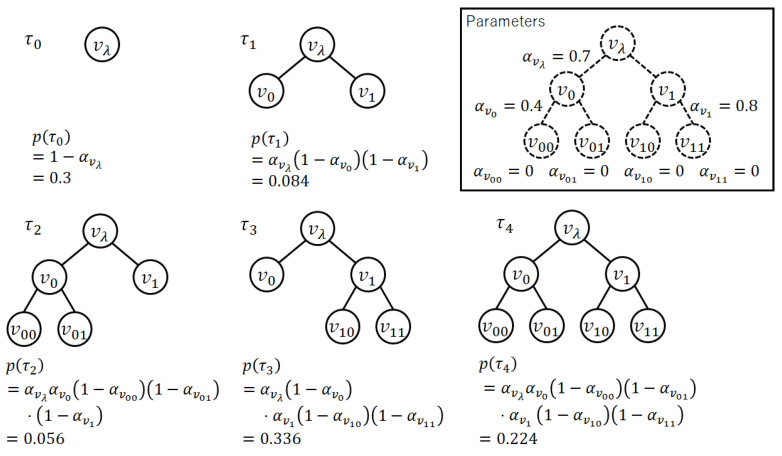
An example of the probability distribution on full rooted subtrees. Here, k=2 and $d_{\max}=2$. The parameters of the distribution are in the upper right figure. When k=2 and $d_{\max}=2$, |T|=5. The probability of each full rooted subtree is calculated under the graph of it.

**Example** **1.** *An example of the probability distribution on full rooted subtrees for k=2 and $d_{\max}=2$ is shown in Figure 2.*

**Theorem** **1.** *The quantity p(τ) defined as in (1) fulfills the condition of the probability distribution, that is, $\sum_{\tau \in T}p\left(\tau \right)=1$.*

**Example** **2.** *Before the proof of Theorem 1 for the general case, we describe an example where $d_{\max}=2$ and k=2 (see Figure 2). First, we factorize the sum as below.*

(2) $$\begin{array}{cc} \; & \sum_{\tau \in T}p\left(\tau \right)\\ \; & =(1-\alpha_{v_{\lambda }})\\ \; & \;+\alpha_{v_{\lambda }}\left(1-\alpha_{v_{0}}\right)\left(1-\alpha_{v_{1}}\right)\\ \; & \;+\alpha_{v_{\lambda }}\alpha_{v_{0}}\left(1-\alpha_{v_{00}}\right)\left(1-\alpha_{v_{01}}\right)\left(1-\alpha_{v_{1}}\right)\\ \; & \;+\alpha_{v_{\lambda }}\left(1-\alpha_{v_{0}}\right)\alpha_{v_{1}}\left(1-\alpha_{v_{10}}\right)\left(1-\alpha_{v_{11}}\right)\\ \; & \;+\alpha_{v_{\lambda }}\alpha_{v_{0}}\left(1-\alpha_{v_{00}}\right)\left(1-\alpha_{v_{01}}\right)\alpha_{v_{1}}\left(1-\alpha_{v_{10}}\right)\left(1-\alpha_{v_{11}}\right) \end{array}$$

(3) $$\begin{array}{cc} \; & =(1-\alpha_{v_{\lambda }})\\ \; & \;+\alpha_{v_{\lambda }}\{\left(1-\alpha_{v_{0}}\right)\left(1-\alpha_{v_{1}}\right)\\ \; & \;\;+\alpha_{v_{0}}\left(1-\alpha_{v_{00}}\right)\left(1-\alpha_{v_{01}}\right)\left(1-\alpha_{v_{1}}\right)\\ \; & \;\;+\left(1-\alpha_{v_{0}}\right)\alpha_{v_{1}}\left(1-\alpha_{v_{10}}\right)\left(1-\alpha_{v_{11}}\right)\\ \; & \;\;+\alpha_{v_{0}}\left(1-\alpha_{v_{00}}\right)\left(1-\alpha_{v_{01}}\right)\alpha_{v_{1}}\left(1-\alpha_{v_{10}}\right)\left(1-\alpha_{v_{11}}\right)\} \end{array}$$

(4) $$\begin{array}{cc} \; & =(1-\alpha_{v_{\lambda }})\\ \; & \;+\alpha_{v_{\lambda }}\{\left(1-\alpha_{v_{0}}\right)\left[\left(1-\alpha_{v_{1}}\right)+\alpha_{v_{1}}\left(1-\alpha_{v_{10}}\right)\left(1-\alpha_{v_{11}}\right)\right]\\ \; & \;\;\;+\alpha_{v_{0}}\left(1-\alpha_{v_{00}}\right)\left(1-\alpha_{v_{01}}\right)\left[\left(1-\alpha_{v_{1}}\right)+\alpha_{v_{1}}\left(1-\alpha_{v_{10}}\right)\left(1-\alpha_{v_{11}}\right)\right]\} \end{array}$$

(5) $$\begin{array}{cc} \; & =(1-\alpha_{v_{\lambda }})\\ \; & \;+\alpha_{v_{\lambda }}\{\left[\left(1-\alpha_{v_{0}}\right)+\alpha_{v_{0}}\left(1-\alpha_{v_{00}}\right)\left(1-\alpha_{v_{01}}\right)\right]\\ \; & \;\;\;\times \left[\left(1-\alpha_{v_{1}}\right)+\alpha_{v_{1}}\left(1-\alpha_{v_{10}}\right)\left(1-\alpha_{v_{11}}\right)\right]\}. \end{array}$$

*Here, $\alpha_{v_{00}}=\alpha_{v_{01}}=\alpha_{v_{10}}=\alpha_{v_{11}}=0$ since $v_{00},v_{01},v_{10},v_{11}\in L_{p}$. Then,*

(6) $$\begin{array}{cc} \sum_{\tau \in T}p\left(\tau \right) & =\left(1-\alpha_{v_{\lambda }}\right)+\alpha_{v_{\lambda }}\left\{\left[\left(1-\alpha_{v_{0}}\right)+\alpha_{v_{0}}\right]\cdot \left[\left(1-\alpha_{v_{1}}\right)+\alpha_{v_{1}}\right]\right\} \end{array}$$

(7) $$\begin{array}{cc} \; & =\left(1-\alpha_{v_{\lambda }}\right)+\alpha_{v_{\lambda }} \end{array}$$

(8) $$\begin{array}{cc} \; & =1. \end{array}$$

The general proof of Theorem 1 is in the following. That also consists of two parts, namely, factorization and substitution. We will first prove Lemma 1, which is the essential lemma, since it is not used only in the proof of Theorem 1 but also in the proof of other theorems later.

**Lemma** **1.** *Let F:T→R be a real-valued function on the set T of the full rooted subtrees of the base tree $\tau_{p}$. If F has the form*

(9) $$\begin{array}{c} F\left(\tau \right)=\prod_{v\in I_{\tau }}G\left(v\right)\prod_{v^{\prime }\in L_{\tau }}H\left(v^{\prime }\right), \end{array}$$

*where $G:V_{p}\to R$ and $H:V_{p}\to R$ are real-valued functions on $V_{p}$, then the summation $\sum_{\tau \in T}F\left(\tau \right)$ can be recursively decomposed as follows.*

(10) $$\begin{array}{c} \sum_{\tau \in T}F\left(\tau \right)=\phi \left(v_{\lambda }\right), \end{array}$$

*where $\phi :V_{p}\to R$ is defined as*

(11) $$\begin{array}{c} \phi \left(v\right):=\left\{\begin{array}{cc} H(v), & v\in L_{p},\\ H\left(v\right)+G\left(v\right)\prod_{v^{\prime }\in {\mathrm{Ch}}_{p}\left(v\right)}\phi \left(v^{\prime }\right), & v\in I_{p}. \end{array}\right. \end{array}$$

**Proof.**  Let $\left[v_{\lambda }\right]$ denote the tree that consists of only the root node $v_{\lambda }$ of the base tree $\tau_{p}$. Then, the cases of the sum is divided as follows.

(12) $$\begin{array}{cc} \sum_{\tau \in T}F\left(\tau \right) & =\sum_{\tau \in T}\prod_{v\in I_{\tau }}G\left(v\right)\prod_{v^{\prime }\in L_{\tau }}H\left(v^{\prime }\right) \end{array}$$

(13) $$\begin{array}{cc} \; & =\prod_{v\in I_{\left[v_{\lambda }\right]}}G\left(v\right)\prod_{v^{\prime }\in L_{\left[v_{\lambda }\right]}}H\left(v^{\prime }\right)+\sum_{\tau \in T\setminus \{\left[v_{\lambda }\right]\}}\prod_{v\in I_{\tau }}G\left(v\right)\prod_{v^{\prime }\in L_{\tau }}H\left(v^{\prime }\right) \end{array}$$

(14) $$\begin{array}{cc} \; & =H\left(v_{\lambda }\right)+\sum_{\tau \in T\setminus \{\left[v_{\lambda }\right]\}}\prod_{v\in I_{\tau }}G\left(v\right)\prod_{v^{\prime }\in L_{\tau }}H\left(v^{\prime }\right) \end{array}$$

(15) $$\begin{array}{cc} \; & =H\left(v_{\lambda }\right)+G\left(v_{\lambda }\right)\sum_{\tau \in T\setminus \{\left[v_{\lambda }\right]\}}\prod_{v\in I_{\tau }\setminus \left\{v_{\lambda }\right\}}G\left(v\right)\prod_{v^{\prime }\in L_{\tau }}H\left(v^{\prime }\right), \end{array}$$

where (14) is because $\left[v_{\lambda }\right]$ has no inner node and its leaf node is only $v_{\lambda }$; (15) is because every tree in $T\setminus [v_{\lambda }]$ has $v_{\lambda }$ and the corresponding factor $G(v_{\lambda })$.

**Figure 3:**
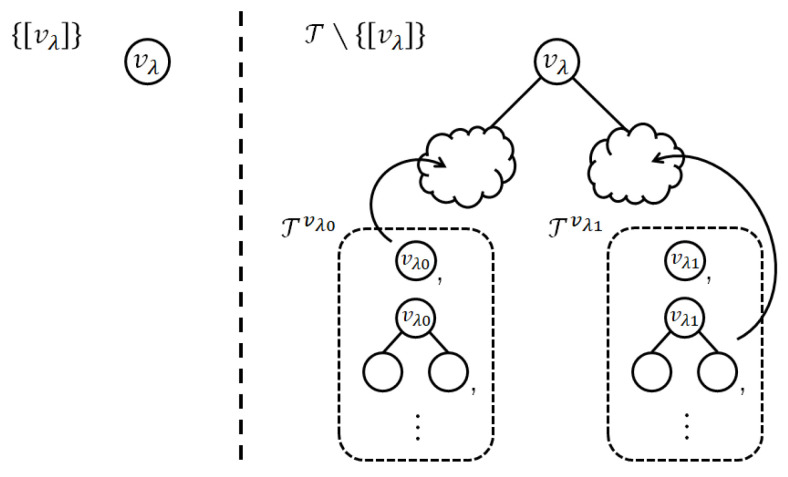
Examples of trees in $\left\{\left[v_{\lambda }\right]\right\}$ and $T\setminus \{\left[v_{\lambda }\right]\}$, where k=2. The left side shows the structure of the tree in $\left\{\left[v_{\lambda }\right]\right\}$. There is only one tree $\left[v_{\lambda }\right]$. The right side shows the structure of the trees in $T\setminus \{\left[v_{\lambda }\right]\}$. All of them have the root node $v_{\lambda }$ as their respective inner nodes. The other structure is determined by choosing subtrees from $T^{v_{\lambda 0}}$ and $T^{v_{\lambda 1}}$.

We have already pointed out that each tree $\tau \in T\setminus \{\left[v_{\lambda }\right]\}$ contains $v_{\lambda }$ as its inner node. The other structure of τ is determined by the shape of *k* subtrees whose root nodes are the child nodes of $v_{\lambda }$ (see Figure 3). We index them in an appropriate order. Then, let $v_{\lambda i}$ denote the *i*-th child node of $v_{\lambda }$ for i∈{0,1,…,k−1}; i.e., $\left\{v_{\lambda 0},\ldots ,v_{\lambda \;k-1}\right\}={\mathrm{Ch}}_{p}\left(v_{\lambda }\right)$. Let $T^{v_{\lambda i}}$ denote the set of subtrees whose root node is $v_{\lambda i}$. Then, there is a natural bijection from $T\setminus \{\left[v_{\lambda }\right]\}$ to $T^{v_{\lambda 0}}\times \cdots \times T^{v_{\lambda \;k-1}}$.

Therefore, the summation of (15) is further factorized. Consequently, we have

$$\begin{array}{cc} \sum_{\tau \in T} & F(\tau )=H\left(v_{\lambda }\right)+G\left(v_{\lambda }\right) \end{array}$$

(16) $$\begin{array}{cc} \; & \times \sum_{\left(\tau_{0},\cdots ,\tau_{k\;-\;1}\right)\in T^{v_{\lambda 0}}\times \cdots \times T^{v_{\lambda \;k\;-\;1}}}\;\left[\prod_{v\in I_{\tau_{0}}}G\left(v\right)\prod_{v^{\prime }\in L_{\tau_{0}}}H\left(v^{\prime }\right)\ldots \prod_{v\in I_{\tau_{k\;-\;1}}}G\left(v\right)\prod_{v^{\prime }\in L_{\tau_{k\;-\;1}}}H\left(v^{\prime }\right)\right]\\ = & H\left(v_{\lambda }\right)+G\left(v_{\lambda }\right) \end{array}$$

(17) $$\begin{array}{cc} \; & \times \sum_{\tau_{0}\in T^{v_{\lambda 0}}}\cdots \sum_{\tau_{k\;-\;1}\in T^{v_{\lambda \;k\;-\;1}}}\;\left[\prod_{v\in I_{\tau_{0}}}G\left(v\right)\prod_{v^{\prime }\in L_{\tau_{0}}}H\left(v^{\prime }\right)\cdots \prod_{v\in I_{\tau_{k\;-\;1}}}G\left(v\right)\prod_{v^{\prime }\in L_{\tau_{k\;-\;1}}}H\left(v^{\prime }\right)\right] \end{array}$$

(18) $$\begin{array}{cc} = & H\left(v_{\lambda }\right)+G\left(v_{\lambda }\right)\prod_{i=0}^{k-1}\sum_{\tau \in T^{v_{\lambda i}}}\prod_{v\in I_{\tau }}G\left(v\right)\prod_{v^{\prime }\in L_{\tau }}H\left(v^{\prime }\right). \end{array}$$

Then, from (12) and (18), we have

(19) $$\begin{array}{c} \underset{\left(a\right)}{\underbrace{\sum_{\tau \in T}\prod_{v\in I_{\tau }}G\left(v\right)\prod_{v^{\prime }\in L_{\tau }}H\left(v^{\prime }\right)}}=H\left(v_{\lambda }\right)+G\left(v_{\lambda }\right)\prod_{i=0}^{k-1}\underset{\left(b\right)}{\underbrace{\sum_{\tau \in T^{v_{\lambda i}}}\prod_{v\in I_{\tau }}G\left(v\right)\prod_{v^{\prime }\in L_{\tau }}H\left(v^{\prime }\right)}}. \end{array}$$

The underbraced parts (a) and (b) have the same structure except for the depth of the root node of the subtree. Therefore, (b) can be decomposed in a similar manner from (12) to (18). We can continue this decomposition to the leaf nodes.

Then, let $T^{v}$ denote the set of subtrees whose root node is $v\in V_{p}$ in general; i.e., we define a notion similar to $T^{v_{\lambda i}}$ for not only $v_{\lambda 0},v_{\lambda 1},\ldots ,v_{\lambda \;k-1}$ but also any other nodes $v\in V_{p}$. Finally, we have an alternative definition of $\phi \left(v\right):V_{p}\to R$, which is equivalent to (11).

(20) $$\begin{array}{c} \phi \left(v\right):=\sum_{\tau \in T^{v}}\prod_{v^{\prime }\in I_{\tau }}G\left(v^{\prime }\right)\prod_{v^{\prime \prime }\in L_{\tau }}H\left(v^{\prime \prime }\right). \end{array}$$

The equivalence is confirmed by substituting it into both sides of (19). Therefore, Lemma 1 is proved. □

Then, the proof of Theorem 1 is as follows.

**Proof of Theorem** **1.** Using Lemma 1, we can divide the cases of the sum and factorize the common terms of $\sum_{\tau \in T}p\left(\tau \right)$ in the following recursive manner.

(21) $$\begin{array}{c} \sum_{\tau \in T}p\left(\tau \right)=\phi \left(v_{\lambda }\right), \end{array}$$

where

(22) $$\begin{array}{c} \phi \left(v\right):=\left\{\begin{array}{cc} 1-\alpha_{v}, & v\in L_{p},\\ \left(1-\alpha_{v}\right)+\alpha_{v}\prod_{v^{\prime }\in {\mathrm{Ch}}_{p}\left(v\right)}\phi \left(v^{\prime }\right), & v\in I_{p}. \end{array}\right. \end{array}$$

Then, we prove ϕ(v)=1 for any node $v\in V_{p}$ by structural induction. For any leaf node $v\in L_{p}$, $\alpha_{v}=0$ from Definition 1. Therefore,

(23) $$\begin{array}{c} \phi \left(v\right)=1-\alpha_{v}=1,\;v\in L_{p}. \end{array}$$

For any inner node $v\in I_{p}$, assuming $\phi (v^{\prime })=1$ as the induction hypothesis for any descendant nodes $v^{\prime }\in {\mathrm{De}}_{p}\left(v\right)$,

(24) $$\begin{array}{cc} \phi (v) & =\left(1-\alpha_{v}\right)+\alpha_{v}\prod_{v^{\prime }\in {\mathrm{Ch}}_{p}\left(v\right)}\phi \left(v^{\prime }\right) \end{array}$$

(25) $$\begin{array}{cc} \; & =\left(1-\alpha_{v}\right)+\alpha_{v}\prod_{v^{\prime }\in {\mathrm{Ch}}_{p}\left(v\right)}1 \end{array}$$

(26) $$\begin{array}{cc} \; & =1. \end{array}$$

Therefore, $\sum_{\tau \in T}p\left(\tau \right)=\phi \left(v_{\lambda }\right)=1$ since $v_{\lambda }$ is also in $V_{p}$. □

**Remark** **1.** *Although Theorem 1 is also proved in [12,13], we extract the essential part of them as Lemma 1. In [10,11], a restricted case of Theorem 1 is proved, in which $\alpha_{v}$ has a common value for all $v\in I_{p}$.*

## 4. Properties of Probability Distribution on Full Rooted Subtrees

In this section, we describe properties of the probability distribution on full rooted subtrees and methods to calculate them. All the proofs are in Appendix A. Note that the motivation and usefulness of Conditions 1, 2, 3, and 4 in this section will be described in Section 5.

### 4.1. Probability of Events on Nodes

At the beginning, we explain why $v\in V_{T}$ determines a probabilistic event. We consider any $v\in V_{p}$ is given as a non-stochastic constant and fixed. After that, a full rooted subtree is randomly chosen according to the probability distribution proposed in Section 3. Then, $V_{T}$ sometimes contains *v* and sometimes not, depending on the realization τ of random variable *T*. Therefore, $v\in V_{T}$ determines a probabilistic event on p(τ). Although the probability of such events are trivially represented as $\sum_{\tau \in T}I\left\{v\in V_{\tau }\right\}p\left(\tau \right)$, where I{·} denotes the indicator function, we derive computationally efficient forms without the summation about τ in the following.

**Theorem** **2.** *For any $v\in V_{p}$, we have the following:*

(27) $$\begin{array}{cc} \; & \mathrm{Pr}\left\{v\in V_{T}\right\}=\prod_{v^{\prime }\in \mathrm{An}\left(v\right)}\alpha_{v^{\prime }}, \end{array}$$

(28) $$\begin{array}{cc} \; & \mathrm{Pr}\left\{v\in I_{T}\right\}=\alpha_{v}\prod_{v^{\prime }\in \mathrm{An}\left(v\right)}\alpha_{v^{\prime }}, \end{array}$$

(29) $$\begin{array}{cc} \; & \mathrm{Pr}\left\{v\in L_{T}\right\}=\left(1-\alpha_{v}\right)\prod_{v^{\prime }\in \mathrm{An}\left(v\right)}\alpha_{v^{\prime }}. \end{array}$$

**Example** **3.** *Let us consider p(τ) shown in Figure 2. Trivially, $\mathrm{Pr}\{v_{01}\in V_{T}\}$, $\mathrm{Pr}\{v_{1}\in I_{T}\}$, and $\mathrm{Pr}\{v_{0}\in L_{T}\}$ are calculated as*

(30) $$\begin{array}{cc} \; & \mathrm{Pr}\left\{v_{01}\in V_{T}\right\}=p\left(\tau_{2}\right)+p\left(\tau_{4}\right)=0.28, \end{array}$$

(31) $$\begin{array}{cc} \; & \mathrm{Pr}\left\{v_{1}\in I_{T}\right\}=p\left(\tau_{3}\right)+p\left(\tau_{4}\right)=0.56, \end{array}$$

(32) $$\begin{array}{cc} \; & \mathrm{Pr}\left\{v_{0}\in L_{T}\right\}=p\left(\tau_{1}\right)+p\left(\tau_{3}\right)=0.42. \end{array}$$

*The same probabilities are also given by*

(33) $$\begin{array}{cc} \; & \mathrm{Pr}\left\{v_{01}\in V_{T}\right\}=\alpha_{v_{\lambda }}\alpha_{v_{0}}=0.28, \end{array}$$

(34) $$\begin{array}{cc} \; & \mathrm{Pr}\left\{v_{1}\in I_{T}\right\}=\alpha_{v_{\lambda }}\alpha_{v_{1}}=0.56, \end{array}$$

(35) $$\begin{array}{cc} \; & \mathrm{Pr}\left\{v_{0}\in L_{T}\right\}=\alpha_{v_{\lambda }}\left(1-\alpha_{v_{1}}\right)=0.42. \end{array}$$

**Remark** **2.** *Probabilities of many other events on nodes are derived from Theorem 2. For example,*

(36) $$\begin{array}{cc} \mathrm{Pr}\{v\in I_{T}|v\in V_{T}\} & =\frac{\mathrm{Pr}\{\left(v\in I_{T}\right)\wedge \left(v\in V_{T}\right)\}}{\mathrm{Pr}\{v\in V_{T}\}} \end{array}$$

(37) $$\begin{array}{cc} \; & =\frac{\mathrm{Pr}\{v\in I_{T}\}}{\mathrm{Pr}\{v\in V_{T}\}} \end{array}$$

(38) $$\begin{array}{cc} \; & =\alpha_{v}. \end{array}$$

### 4.2. Mode

We describe an algorithm to find the mode of p(τ) with $O(k^{d_{\max}+1})$ computational cost. (O(·) denotes the Big-O notation, i.e., f(n)=O(g(n)) means that ${}^{\exists }k>0{,}^{\exists }n_{0}>0{,}^{\forall }n>n_{0},\left|f\left(n\right)\right|\leq k\cdot g\left(n\right)$). Note that the size of search space T is of the order of $\Omega \left(2^{k^{d_{\max}-2}}\right)$ in general. (Ω(·) denotes the Big-Omega notation in complexity theory; i.e., f(n)=Ω(g(n)) means that ${}^{\exists }k>0{,}^{\exists }n_{0}>0{,}^{\forall }n>n_{0},f\left(n\right)\geq k\cdot g\left(n\right)$. $\left|T\right|=\Omega \left(2^{k^{d_{\max}-2}}\right)$ is proved by substituting G(v)≡H(v)≡1 in Lemma 1). First, by replacing all the sum in the proof of Lemma 1 with the maximum, we can derive the following recursive expression of $\max_{\tau \in T}p\left(\tau \right)$.

**Proposition** **1.**
(39) $$\begin{array}{c} \max_{\tau \in T}p\left(\tau \right)=\psi \left(v_{\lambda }\right), \end{array}$$

*where*

(40) $$\begin{array}{c} \psi \left(v\right):=\left\{\begin{array}{cc} 1, & v\in L_{p},\\ \max\left\{1-\alpha_{v},\alpha_{v}\prod_{v^{\prime }\in {\mathrm{Ch}}_{p}\left(v\right)}\psi \left(v^{\prime }\right)\right\}, & v\in I_{p}. \end{array}\right. \end{array}$$

**Example** **4.** *On p(τ) shown in Figure 2, the maximum probability is $p(\tau_{3})=0.336$. It is also calculated as follows.*

(41) $$\begin{array}{cc} \; & \max\{1-\alpha_{v_{\lambda }},\alpha_{v_{\lambda }}\max\left\{1-\alpha_{v_{0}},\alpha_{v_{0}}\right\}\cdot \max\left\{1-\alpha_{v_{1}},\alpha_{v_{1}}\right\}\} \end{array}$$

(42) $$\begin{array}{cc} \; & =\max\{0.3,0.7\max\{0.6,0.4\}\cdot \max\{0.2,0.8\}\} \end{array}$$

(43) $$\begin{array}{cc} \; & =\max\{0.3,0.336\}=0.336. \end{array}$$

In addition, we define a flag variable $\delta_{v}\in \left\{0,1\right\}$ as follows.

**Definition** **2.** *For any $v\in V_{p}$, we define*

(44) $$\begin{array}{c} \delta_{v}:=\left\{\begin{array}{cc} 1, & 1-\alpha_{v}<\alpha_{v}\prod_{v^{\prime }\in {\mathrm{Ch}}_{p}\left(v\right)}\psi \left(v^{\prime }\right),\\ 0, & \mathrm{otherwise}. \end{array}\right. \end{array}$$

We can calculate ψ(v) and $\delta_{v}$ simultaneously. Then, the mode of p(τ) is given by the following proposition.

**Proposition** **2.** *$\arg\max_{\tau \in T}p\left(\tau \right)$ is identified as the tree that satisfies*

(45) $$\begin{array}{c} v\in I_{\tau }\Rightarrow \delta_{v}=1, \end{array}$$

(46) $$\begin{array}{c} v\in L_{\tau }\Rightarrow \delta_{v}=0. \end{array}$$

Then, the following theorem holds.

**Theorem** **3.** *The mode of p(τ) can be found via backtracking search from $v_{\lambda }$ after the calculation of ψ(v) and $\delta_{v}$. It is detailed in Algorithm A1 in Appendix B.*

**Remark** **3.** *In [10,11], Papageorgiou et al. proposed the same algorithm as Algorithm A1 and an algorithm to find multiple most likely trees on the background of text compression.*

**Figure 4:**
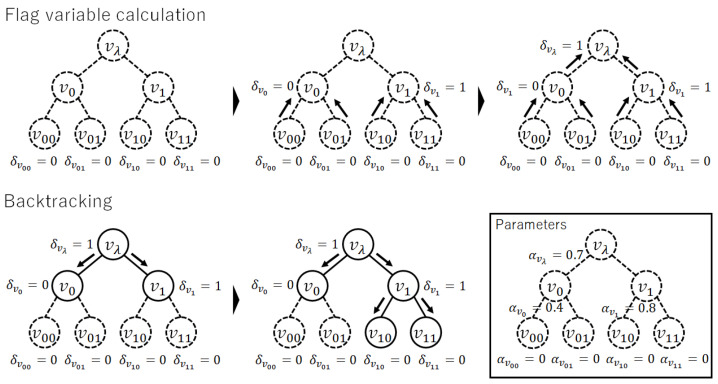
An example of the mode calculation. The parameters are the same as those in Figure 2 and are shown in the lower right diagram. Diagrams in the top half show the process of calculation for the flag variable $\delta_{v}$, which is determined from leaf nodes in order. Diagrams in the lower half show the process of backtracking. If $\delta_{v}=1$, expand the edge. If $\delta_{v}=0$, stop the expansion.

**Example** **5.** *See Figure 4. The parameters are the same as those in Figure 2. The mode $\tau_{3}$ is found by the proposed algorithm.*

### 4.3. Expectation

Let f:T→R denote a real-valued function on T. Here, we discuss sufficient conditions of *f*, under which the following expectation can be calculated efficiently with $O(k^{d_{\max}+1})$ cost.

(47) $$\begin{array}{c} E\left[f\left(T\right)\right]:=\sum_{\tau \in T}f\left(\tau \right)p\left(\tau \right). \end{array}$$

Note that the size of T is of the order of $\Omega \left(2^{k^{d_{\max}-2}}\right)$ in general.

**Condition** **1.** *There exist $g:V_{p}\to R$ and $h:V_{p}\to R$ such that*

(48) $$\begin{array}{c} f\left(\tau \right)=\prod_{v\in I_{\tau }}g\left(v\right)\prod_{v^{\prime }\in L_{\tau }}h\left(v^{\prime }\right). \end{array}$$

**Theorem** **4.** *Under Condition 1, we define a recursive function $\phi :V_{p}\to R$ as*

(49) $$\begin{array}{c} \phi \left(v\right):=\left\{\begin{array}{cc} h(v), & v\in L_{p},\\ \left(1-\alpha_{v}\right)h\left(v\right)+\alpha_{v}g\left(v\right)\prod_{v^{\prime }\in {\mathrm{Ch}}_{p}\left(v\right)}\phi \left(v^{\prime }\right), & v\in I_{p}. \end{array}\right. \end{array}$$

*Then, we can calculate E[f(T)] as $E\left[f\left(T\right)\right]=\phi \left(v_{\lambda }\right)$.*

**Example** **6.** *Theorem 2 can be regarded examples of Theorem 4.*

**Condition** **2.** *There exist $g:V_{p}\to R$ and $h:V_{p}\to R$ such that*

(50) $$\begin{array}{c} f\left(\tau \right)=\sum_{v\in I_{\tau }}g\left(v\right)+\sum_{v^{\prime }\in L_{\tau }}h\left(v^{\prime }\right). \end{array}$$

**Theorem** **5.** *Under Condition 2, we define a recursive function $\xi :V_{p}\to R$ as*

(51) $$\begin{array}{c} \xi \left(v\right):=\left\{\begin{array}{cc} h(v), & v\in L_{p},\\ \left(1-\alpha_{v}\right)h\left(v\right)+\alpha_{v}\left(g\left(v\right)+\sum_{v^{\prime }\in {\mathrm{Ch}}_{p}\left(v\right)}\xi \left(v^{\prime }\right)\right), & v\in I_{p}. \end{array}\right. \end{array}$$

*Then, we can calculate E[f(T)] as $E\left[f\left(T\right)\right]=\xi \left(v_{\lambda }\right)$.*

**Remark** **4.** *Theorem 5 is useful to calculate the Shannon entropy (see, e.g., [16]) of p(τ). It is described in Section 4.4.*

### 4.4. Shannon Entropy

**Corollary** **1.** *By substituting $g\left(v\right)=-\log\alpha_{v}$ and $h\left(v\right)=-\log\left(1-\alpha_{v}\right)$ into (51), the Shannon entropy $H\left[T\right]:=-\sum_{\tau \in T}p\left(\tau \right)\log p\left(\tau \right)$ can be recursively calculated as follows.*

(52) $$\begin{array}{c} H\left[T\right]=\xi \left(v_{\lambda }\right), \end{array}$$

*where*

(53) $$\begin{array}{c} \xi \left(v\right):=\left\{\begin{array}{cc} 0, & v\in L_{p},\\ -\left(1-\alpha_{v}\right)\log\left(1-\alpha_{v}\right)+\alpha_{v}\left(-\log\alpha_{v}+\sum_{v^{\prime }\in {\mathrm{Ch}}_{p}\left(v\right)}\xi \left(v^{\prime }\right)\right), & v\in I_{p}. \end{array}\right. \end{array}$$

**Remark** **5.** *Kullback–Leibler divergence (see, e.g., [16]) between two tree distributions p(τ) and $p^{\prime }\left(\tau \right)$ can be calculated in a similar manner to Corollary 1. This fact may be useful for variational Bayesian inference, in which the Kullback–Leibler divergence is minimized. This will be future work.*

### 4.5. Conjugate Prior for a Probability Distribution on Full Rooted Subtrees

Here, we consider that $\alpha_{v}\in \left[0,1\right]$ is also a realization of a random variable. Let α denote ${\left\{\alpha_{v}\right\}}_{v\in V_{p}}$, and we describe p(τ) as p(τ|α) to emphasize the dependency of α in the following theorem. Then, a conjugate prior for p(τ|α) is as follows.

**Theorem** **6.** *The following probability distribution is a conjugate prior for p(τ|α).*

(54) $$\begin{array}{c} p\left(\alpha \right):=\prod_{v\in V_{p}}\mathrm{Beta}\left(\alpha_{v}|\beta_{v},\gamma_{v}\right), \end{array}$$

*where $\mathrm{Beta}(\cdot |\beta_{v},\gamma_{v})$ denotes the probability density function of the beta distribution whose parameters are $\beta_{v}$ and $\gamma_{v}$. More precisely,*

(55) $$\begin{array}{c} p\left(\alpha |\tau \right)=\prod_{v\in V_{p}}\mathrm{Beta}\left(\alpha_{v}|\beta_{v|\tau },\gamma_{v|\tau }\right), \end{array}$$

*where*

(56) $$\begin{array}{cc} \beta_{v|\tau }:= & \left\{\begin{array}{cc} \beta_{v}+1, & v\in I_{\tau },\\ \beta_{v}, & \mathrm{otherwise}, \end{array}\right. \end{array}$$

(57) $$\begin{array}{cc} \gamma_{v|\tau }:= & \left\{\begin{array}{cc} \gamma_{v}+1, & v\in L_{\tau },\\ \gamma_{v}, & \mathrm{otherwise}. \end{array}\right. \end{array}$$

### 4.6. Probability Distribution on a Full Rooted Tree as a Conjugate Prior

We define another random variable *X* on a set X and assume *X* depends on *T*; i.e., it follows a distribution p(x|τ). Here, we discuss a sufficient condition of p(x|τ), under which p(τ) becomes a conjugate prior for it, and we can efficiently calculate the posterior p(τ|x).

**Condition** **3.** *There exist two functions $g:V_{p}\times X\to R$ and $h:V_{p}\times X\to R$, and p(x|τ) has the following form.*

(58) $$\begin{array}{c} p\left(x|\tau \right)=\prod_{v\in I_{\tau }}g\left(x,v\right)\prod_{v^{\prime }\in L_{\tau }}h\left(x,v^{\prime }\right). \end{array}$$

*Note that g and h are not necessarily probability density functions.*

**Example** **7.** *For given $\mu_{1},\mu_{2}\in R$ and $\sigma_{1},\sigma_{2}\in R_{>0}$, let $N(x|\mu_{1},\sigma_{1}^{2})$ and $N(x|\mu_{2},\sigma_{2}^{2})$ denote the probability density functions of the normal distributions governed by them. Let $x:={\left(x_{v}\right)}_{v\in V_{p}}$. If we assume*

(59) $$\begin{array}{cc} \; & g\left(x,v\right)=N\left(x_{v}|\mu_{1},\sigma_{1}^{2}\right), \end{array}$$

(60) $$\begin{array}{cc} \; & h\left(x,v\right)=\prod_{v^{\prime }\in {\mathrm{De}}_{p}\left(v\right)\cup \left\{v\right\}}N\left(x_{v^{\prime }}|\mu_{2},\sigma_{2}^{2}\right), \end{array}$$

*we can construct p(x|τ) that satisfies Condition 3. In other words, the elements of the $|V_{p}|$ dimensional vector x follow the mixture of two normal distributions, and either of the two is chosen by τ.*

**Theorem** **7.** *Under Condition 3, we define q(x|v):= and $\alpha_{v|x}$ as follows.*

(61) $$\begin{array}{cc} q(x|v):= & \left\{\begin{array}{cc} h(x,v), & v\in L_{p},\\ \left(1-\alpha_{v}\right)h\left(x,v\right)+\alpha_{v}g\left(x,v\right)\prod_{v^{\prime }\in {\mathrm{Ch}}_{p}\left(v\right)}q\left(x|v^{\prime }\right), & v\in I_{p}, \end{array}\right. \end{array}$$

(62) $$\begin{array}{cc} \alpha_{v|x}:= & \left\{\begin{array}{cc} \alpha_{v}, & v\in L_{p},\\ \frac{\alpha_{v}g\left(x,v\right)\prod_{v^{\prime }\in {\mathrm{Ch}}_{p}\left(v\right)}q\left(x|v^{\prime }\right)}{q(x|v)}, & v\in I_{p}. \end{array}\right. \end{array}$$

*Note that $\alpha_{v}=0$ for $v\in L_{p}$ (see Definition 1). Then, the posterior p(τ|x) is represented as follows.*

(63) $$\begin{array}{c} p\left(\tau |x\right)=\prod_{v\in I_{\tau }}\alpha_{v|x}\prod_{v^{\prime }\in L_{\tau }}\left(1-\alpha_{v^{\prime }|x}\right). \end{array}$$

It should be noted that the calculation of q(x|v) and $\alpha_{v|x}$ requires $O(k^{d_{\max}+1})$ cost, and it requires $\Omega \left(2^{k^{d_{\max}-2}}\right)$ cost in general.

Moreover, if we assume the following condition to be stronger than Condition 3, we can calculate the posterior p(τ|x) more efficiently with $O(d_{\max})$ cost.

**Condition** **4.** *In addition to Condition 3, we assume that there exists a path from $v_{\lambda }$ to a leaf node $v_{\mathrm{end}}\in L_{p}$ and another function $h^{\prime }:V_{p}\times X\to R$, which satisfy*

(64) $$\begin{array}{cc} g(x,v) & \equiv 1, \end{array}$$

(65) $$\begin{array}{cc} h(x,v):= & h^{\prime }{\left(x,v\right)}^{I\{v⪰v_{\mathrm{end}}\}}. \end{array}$$

*Here, I{·} denotes the indicator function. In other words, only h(x,v) on the path from $v_{\lambda }$ to $v_{\mathrm{end}}$ takes a value different of 1.*

**Corollary** **2.** *Under Condition 4, q(x|v) and $\alpha_{v|x}$ are calculated as follows, more efficiently than (61) and (62).*

(66) $$\begin{array}{cc} q(x|v) & =\left\{\begin{array}{cc} h^{\prime }\left(x,v\right), & v=v_{\mathrm{end}},\\ \left(1-\alpha_{v}\right)h^{\prime }\left(x,v\right)+\alpha_{v}q\left(x|v_{\mathrm{ch}}\right), & v≻v_{\mathrm{end}},\\ 1, & \mathrm{otherwise}, \end{array}\right. \end{array}$$

(67) $$\begin{array}{cc} \alpha_{v|x} & =\left\{\begin{array}{cc} \alpha_{v}, & v⊁v_{\mathrm{end}},\\ \frac{\alpha_{v}q\left(x|v_{\mathrm{ch}}\right)}{q(x|v)}, & v≻v_{\mathrm{end}}, \end{array}\right. \end{array}$$

*where $v_{\mathrm{ch}}$ is a child node of v on the path from $v_{\lambda }$ to $v_{\mathrm{end}}$. Note that we need not calculate q(x|v) for $v⋡v_{\mathrm{end}}$ to update the posterior, and it costs only $O(d_{\max})$.*

**Remark** **6.** *Condition 4 is effective at representing the generation of sequential data $x_{1},x_{2},\ldots ,x_{N}$, in which there exists a path from root node $v_{\lambda }$ to a leaf node $v_{\mathrm{end}}^{n}\in L_{p}$ for each n∈{1,2,…,N} ($v_{\mathrm{end}}^{n}$ and $v_{\mathrm{end}}^{n^{\prime }}$ may different each other for $n\neq n^{\prime }$). The remarkable previous studies using Corollary 2 are [8,9,10,11,12,13] (In [10,11], only (66) is used but (67) is not). In other words, they treat only the case under Condition 4. The other theorems in this paper have potential applications to broader fields of study.*

## 5. Discussion

In this section, we describe the usefulness of our results in statistical decision theory (see, e.g., [6]) and hierarchical Bayesian modeling (see, e.g., [15]). First, our results are useful in model selection and model averaging under the Bayes criterion in statistical decision theory (see, e.g., [6]). The proposed probability distribution p(τ) is a conjugate prior for stochastic models p(x|τ) satisfying Condition 3, as shown in Theorem 7, and the MAP estimate $\arg\max_{\tau }p\left(\tau |x\right)$ can be efficiently calculated by applying Theorem 3 to the posterior distribution p(τ|x) obtained by Theorem 7. This is the Bayes optimal model selection based on the posterior distribution. Furthermore, we can calculate $\sum_{\tau }p\left(x_{\mathrm{new}}|\tau \right)p\left(\tau |x\right)$, i.e., the weighting of the stochastic models based on the posterior distribution, by using Theorems 4 and 7, since the stochastic models p(x|τ) satisfying Condition 3 also satisfy Condition 1. This is model averaging of all possible trees with Bayes optimal weights. This corresponds to the methodologies in which they do not select a single tree but aggregate several trees, such as [4,5]. It should be noted that the occurrence probability of a deep tree exponentially decays in our proposed probability distribution. Therefore, we can avoid the deep tree, which often corresponds to a complex statistical model, as mentioned in Section 1.

Second, one example of the applications derived from our results is hyperparameter learning. As mentioned in Remark 6, Condition 4 has been applied to various stochastic models p(x|τ) in previous studies [8,9,10,11,12,13]. Conditions 1 and 3 are more generalized conditions than Condition 4, since the stochastic model p(x|τ) satisfying Condition 4 also satisfies Conditions 1 and 3. In addition, the logarithm of a function f(τ) satisfying Conditions 1 and 3 (and a stochastic model p(x|τ) satisfying Condition 4) satisfies Condition 2. Therefore, we can calculate $\sum_{\tau \in T}p\left(\tau |x\right)\log p\left(x|\tau \right)$ by using Theorems 7 and 5. In particular, the fact that we can calculate the expectations $E\left[p\left(x|T\right)\right]=\sum_{\tau \in T}p\left(\tau |x\right)p\left(x|\tau \right)$ and $E\left[\log p\left(x|T\right)\right]=\sum_{\tau \in T}p\left(\tau |x\right)\log p\left(x|\tau \right)$ of the stochastic model p(x|τ) satisfying Condition 4 implies that we can learn hyperparameters of the stochastic models in [8,9,10,11,12,13] by hierarchical Bayesian modeling with variational Bayesian methods (see, e.g., [15]). To the best of our knowledge, there are no unified studies treating hyperparameter learning for these models.

## 6. Future Work

Since the present study is a theoretical study, the theorems derived will be applied in future studies. Theorems 1 and 3 and Corollary 2 have been used in previous studies [8,9,10,11,12,13]. Therefore, the other theorems can be applied.

In this study, we did not use approximative algorithms such as the variational Bayes or Markov chain Monte Carlo method (see, e.g., [15]). Such algorithms are required for learning hierarchical models that contain the probability distribution on full rooted subtrees. The methods proposed herein may serve as subroutines. The expansion of our methods to approximative algorithms is another future work.

In this study, the class of trees is restricted to that of full trees, in which every inner node has the same number of child nodes. Hence, another the generalization of the class to that of any rooted tree can be considered in future studies.

## 7. Conclusions

In this paper, we discussed the probability distributions on full rooted subtrees. Although such a distribution has been used in many fields of study, such as information theory [8,9,10,11], image processing [12], and machine learning [13], it depends significantly on the specific applications and data generative models. By contrast, we discussed it theoretically, collectively, and independently from a specific data generative model. Subsequently, we derived new generalized methods to evaluate the characteristics of the probability distribution on full rooted subtrees, which have not been performed in previous studies. The derived methods are efficient for calculating the events on the nodes, the mode, the expectation, the Shannon entropy, and the posterior distribution of a full rooted subtree. Therefore, this study expands the possibility of the applying the probability distribution on full rooted subtrees.

## Data Availability

Not applicable.

## Appendix A

**Proof of Theorem** **2.** First, we prove (27). Let I{·} denote the indicator function. Then, $\mathrm{Pr}\{v\in V_{T}\}$ is expressed as

(A1) $$\begin{array}{c} \mathrm{Pr}\left\{v\in V_{T}\right\}=\sum_{\tau \in T}I\left\{v\in V_{\tau }\right\}p\left(\tau \right). \end{array}$$

Here, $v\in V_{\tau }$ is equivalent that all the leaf nodes is not a ancestor node of *v*. Then,

(A2) $$\begin{array}{cc} \mathrm{Pr}\{v\in V_{T}\} & =\sum_{\tau \in T}\prod_{v^{\prime }\in L_{\tau }}I\left\{v^{\prime }⊁v\right\}p\left(\tau \right) \end{array}$$

(A3) $$\begin{array}{cc} \; & =\sum_{\tau \in T}\prod_{v^{\prime }\in I_{\tau }}\alpha_{v^{\prime }}\prod_{v^{\prime \prime }\in L_{\tau }}I\left\{v^{\prime \prime }⊁v\right\}\left(1-\alpha_{v^{\prime \prime }}\right). \end{array}$$

Therefore, using Lemma 1,

(A4) $$\begin{array}{c} \mathrm{Pr}\left\{v\in V_{T}\right\}=\phi_{v}\left(v_{\lambda }\right), \end{array}$$

where

(A5) $$\begin{array}{c} \phi_{v}\left(v^{\prime }\right):=\left\{\begin{array}{cc} I\left\{v^{\prime }⊁v\right\}\left(1-\alpha_{v^{\prime }}\right), & v^{\prime }\in L_{p},\\ I\left\{v^{\prime }⊁v\right\}\left(1-\alpha_{v^{\prime }}\right)+\alpha_{v^{\prime }}\prod_{v^{\prime \prime }\in {\mathrm{Ch}}_{p}\left(v^{\prime }\right)}\phi_{v}\left(v^{\prime \prime }\right), & v^{\prime }\in I_{p}. \end{array}\right. \end{array}$$

We further transform this function. If $v^{\prime }⊁v$, then $I\{v^{\prime }⊁v\}=1$ and consequently,

(A6) $$\begin{array}{c} \phi_{v}\left(v^{\prime }\right)=\left\{\begin{array}{cc} (1-\alpha_{v^{\prime }}), & v^{\prime }\in L_{p},\\ \left(1-\alpha_{v^{\prime }}\right)+\alpha_{v^{\prime }}\prod_{v^{\prime \prime }\in {\mathrm{Ch}}_{p}\left(v^{\prime }\right)}\phi_{v}\left(v^{\prime \prime }\right), & v^{\prime }\in I_{p}. \end{array}\right. \end{array}$$

It has the same form as (22), and every child node $v^{\prime \prime }\in {\mathrm{Ch}}_{p}\left(v^{\prime }\right)$ also satisfies $v^{\prime \prime }⊁v$. Therefore, $\phi_{v}\left(v^{\prime }\right)=1$ for $v^{\prime }⊁v$. If $v^{\prime }≻v$, $v^{\prime }$ cannot be in $L_{p}$ and has only one child node in An(v)∪{v}. Let $v_{\mathrm{ch}}^{\prime }$ denote it. Then, $\phi_{v}\left(v^{\prime \prime }\right)=1$ for the other child nodes $v^{\prime \prime }\in {\mathrm{Ch}}_{p}\left(v^{\prime }\right)\setminus \left\{v_{\mathrm{ch}}^{\prime }\right\}$. Therefore, (A5) is represented as follows.

(A7) $$\begin{array}{c} \phi_{v}\left(v^{\prime }\right)=\alpha_{v^{\prime }}\phi_{v}\left(v_{\mathrm{ch}}^{\prime }\right). \end{array}$$

Therefore, expanding $\phi_{v}\left(v_{\lambda }\right)$,

(A8) $$\begin{array}{c} \mathrm{Pr}\left\{v\in V_{T}\right\}=\prod_{v^{\prime }\in \mathrm{An}\left(v\right)}\alpha_{v^{\prime }}. \end{array}$$

Next, we prove (28). It is proved in a similar manner to the proof of (27) since

(A9) $$\begin{array}{c} \mathrm{Pr}\left\{v\in I_{T}\right\}=\sum_{\tau \in T}\prod_{v^{\prime }\in L_{\tau }}I\left\{v^{\prime }⋡v\right\}p\left(\tau \right). \end{array}$$

Lastly, we prove (29). We have

(A10) $$\begin{array}{c} \mathrm{Pr}\left\{v\in L_{T}\right\}=\mathrm{Pr}\left\{v\in V_{T}\right\}-\mathrm{Pr}\left\{v\in I_{T}\right\}. \end{array}$$

Therefore, (29) follows from (27) and (28). □

**Proof of Theorem** **4.** By substituting (48) into (47), E[f(T)] can be represented as follows.

(A11) $$\begin{array}{c} E\left[f\left(T\right)\right]=\sum_{\tau \in T}\prod_{v\in I_{\tau }}\alpha_{v}g\left(v\right)\prod_{v^{\prime }\in L_{\tau }}\left(1-\alpha_{v^{\prime }}\right)h\left(v^{\prime }\right). \end{array}$$

Then, using Lemma 1, Theorem 4 straightforwardly follows. □

**Proof of Theorem** **5.** First, we switch the order of the summation as follows.

(A12) $$\begin{array}{cc} E[f(T)] & =\sum_{\tau \in T}p\left(\tau \right)\left(\sum_{v\in I_{\tau }}g\left(v\right)+\sum_{v^{\prime }\in L_{\tau }}h\left(v^{\prime }\right)\right) \end{array}$$

(A13) $$\begin{array}{cc} \; & =\sum_{\tau \in T}p\left(\tau \right)\sum_{v\in V_{p}}I\left\{v\in I_{\tau }\right\}g\left(v\right)+\sum_{\tau \in T}p\left(\tau \right)\sum_{v^{\prime }\in V_{p}}I\left\{v^{\prime }\in L_{\tau }\right\}h\left(v^{\prime }\right) \end{array}$$

(A14) $$\begin{array}{cc} \; & =\sum_{v\in V_{p}}g\left(v\right)\sum_{\tau \in T}p\left(\tau \right)I\left\{v\in I_{\tau }\right\}+\sum_{v^{\prime }\in V_{p}}h\left(v^{\prime }\right)\sum_{\tau \in T}p\left(\tau \right)I\left\{v^{\prime }\in L_{\tau }\right\} \end{array}$$

(A15) $$\begin{array}{cc} \; & =\sum_{v\in V_{p}}g\left(v\right)\mathrm{Pr}\left\{v\in I_{T}\right\}+\sum_{v^{\prime }\in V_{p}}h\left(v^{\prime }\right)\mathrm{Pr}\left\{v^{\prime }\in L_{T}\right\} \end{array}$$

(A16) $$\begin{array}{cc} \; & =\sum_{v\in V_{p}}g\left(v\right)\alpha_{v}\prod_{v^{\prime }\in \mathrm{An}\left(v\right)}\alpha_{v^{\prime }}+\sum_{v\in V_{p}}h\left(v\right)\left(1-\alpha_{v}\right)\prod_{v^{\prime }\in \mathrm{An}\left(v\right)}\alpha_{v^{\prime }} \end{array}$$

(A17) $$\begin{array}{cc} \; & =\sum_{v\in V_{p}}\left(\alpha_{v}g\left(v\right)+\left(1-\alpha_{v}\right)h\left(v\right)\right)\prod_{v^{\prime }\in \mathrm{An}\left(v\right)}\alpha_{v^{\prime }}, \end{array}$$

where (A16) is because of Theorem 2. Next, we decompose the right-hand side of (A17) until it has the same form as (51). Since $V_{p}=\left\{v_{\lambda }\right\}\cup {\mathrm{De}}_{p}\left(v_{\lambda }\right)$,

(A18) $$\begin{array}{c} \left(A17\right)=\alpha_{v_{\lambda }}g\left(v_{\lambda }\right)+\left(1-\alpha_{v_{\lambda }}\right)h\left(v_{\lambda }\right)+\sum_{v\in {\mathrm{De}}_{p}\left(v_{\lambda }\right)}\left(\alpha_{v}g\left(v\right)+\left(1-\alpha_{v}\right)h\left(v\right)\right)\prod_{v^{\prime }\in \mathrm{An}\left(v\right)}\alpha_{v^{\prime }}. \end{array}$$

For any $v\in {\mathrm{De}}_{p}\left(v_{\lambda }\right)$, An(v) contains $v_{\lambda }$. Therefore,

(A19) $$\begin{array}{cc} \left(A18\right) & =\left(1-\alpha_{v_{\lambda }}\right)h\left(v_{\lambda }\right)+\alpha_{v_{\lambda }}\left(g\left(v_{\lambda }\right)+\sum_{v\in {\mathrm{De}}_{p}\left(v_{\lambda }\right)}\left(\alpha_{v}g\left(v\right)+\left(1-\alpha_{v}\right)h\left(v\right)\right)\prod_{v^{\prime }:v_{\lambda }≻v^{\prime }≻v}\alpha_{v^{\prime }}\right). \end{array}$$

Further, since ${\mathrm{De}}_{p}\left(v_{\lambda }\right)={⋃}_{v\in {\mathrm{Ch}}_{p}\left(v_{\lambda }\right)}\left({\mathrm{De}}_{p}\left(v\right)\cup \left\{v\right\}\right)$,

(A20) $$\begin{array}{cc} \; & \left(A19\right)=\left(1-\alpha_{v_{\lambda }}\right)h\left(v_{\lambda }\right)\\ \; & \;+\alpha_{v_{\lambda }}\left(g\left(v_{\lambda }\right)+\sum_{v\in {\mathrm{Ch}}_{p}\left(v_{\lambda }\right)}\left[\sum_{v^{\prime }\in {\mathrm{De}}_{p}\left(v\right)\cup \left\{v\right\}}\left(\alpha_{v^{\prime }}g\left(v^{\prime }\right)+\left(1-\alpha_{v^{\prime }}\right)h\left(v^{\prime }\right)\right)\prod_{v^{\prime \prime }:v⪰v^{\prime \prime }≻v^{\prime }}\alpha_{v^{\prime \prime }}\right]\right). \end{array}$$

Comparing (A17) and (A20), we have

(A21) $$\begin{array}{cc} \; & \underset{\left(a\right)}{\underbrace{\sum_{v\in V_{p}}\left(\alpha_{v}g\left(v\right)+\left(1-\alpha_{v}\right)h\left(v\right)\right)\prod_{v^{\prime }\in \mathrm{An}\left(v\right)}\alpha_{v^{\prime }}}}\\ \; & =\left(1-\alpha_{v_{\lambda }}\right)h\left(v_{\lambda }\right)\\ \; & \;+\alpha_{v_{\lambda }}\left(g\left(v_{\lambda }\right)+\sum_{v\in {\mathrm{Ch}}_{p}\left(v_{\lambda }\right)}\underset{\left(b\right)}{\left[\underbrace{\sum_{v^{\prime }\in {\mathrm{De}}_{p}\left(v\right)\cup \left\{v\right\}}\left(\alpha_{v^{\prime }}g\left(v^{\prime }\right)+\left(1-\alpha_{v^{\prime }}\right)h\left(v^{\prime }\right)\right)\prod_{v^{\prime \prime }:v⪰v^{\prime \prime }≻v^{\prime }}\alpha_{v^{\prime \prime }}}\right]}\right). \end{array}$$

The underbraced parts (a) and (b) have the same structure. Therefore, (b) can be decomposed in a similar manner from (A17) to (A20). We can continue this decomposition to the leaf nodes. Finally, we have an alternative definition of $\xi \left(v\right):V_{p}\to R$, which is equivalent to (51).

(A22) $$\begin{array}{cc} \xi (v):= & \sum_{v^{\prime }\in {\mathrm{De}}_{p}\left(v\right)\cup \left\{v\right\}}\left(\alpha_{v^{\prime }}g\left(v^{\prime }\right)+\left(1-\alpha_{v^{\prime }}\right)h\left(v^{\prime }\right)\right)\prod_{v^{\prime \prime }:v⪰v^{\prime \prime }≻v^{\prime }}\alpha_{v^{\prime \prime }}. \end{array}$$

The equivalence is confirmed by substituting it into both sides of (A21). Therefore, Theorem 5 is proved. □

**Proof of Theorem** **6.** By the Bayes theorem, we have

(A23) $$\begin{array}{cc} p(\alpha |\tau ) & \propto p(\tau |\alpha )p(\alpha ) \end{array}$$

(A24) $$\begin{array}{cc} \; & =\prod_{v\in I_{\tau }}\alpha_{v}\prod_{v^{\prime }\in L_{\tau }}\left(1-\alpha_{v^{\prime }}\right)\prod_{v^{\prime \prime }\in V_{p}}\mathrm{Beta}\left(\alpha_{v}^{\prime \prime }|\beta_{v^{\prime \prime }},\gamma_{v^{\prime \prime }}\right) \end{array}$$

(A25) $$\begin{array}{cc} \; & =\prod_{v\in I_{\tau }}\alpha_{v}\mathrm{Beta}\left(\alpha_{v}|\beta_{v},\gamma_{v}\right)\prod_{v^{\prime }\in L_{\tau }}\left(1-\alpha_{v^{\prime }}\right)\mathrm{Beta}\left(\alpha_{v}^{\prime }|\beta_{v^{\prime }},\gamma_{v^{\prime }}\right)\prod_{v^{\prime \prime }\in V_{p}\setminus V_{\tau }}\mathrm{Beta}\left(\alpha_{v}^{\prime \prime }|\beta_{v^{\prime \prime }},\gamma_{v^{\prime \prime }}\right) \end{array}$$

(A26) $$\begin{array}{cc} \; & \propto \prod_{v\in V_{p}}\mathrm{Beta}\left(\alpha_{v}|\beta_{v|\tau },\gamma_{v|\tau }\right), \end{array}$$

where we used the conjugate property between the Bernoulli distribution and the beta distribution for each term and

(A27) $$\begin{array}{cc} \beta_{v|\tau }:= & \left\{\begin{array}{cc} \beta_{v}+1, & v\in I_{\tau },\\ \beta_{v}, & \mathrm{otherwise}, \end{array}\right. \end{array}$$

(A28) $$\begin{array}{cc} \gamma_{v|\tau }:= & \left\{\begin{array}{cc} \gamma_{v}+1, & v\in L_{\tau },\\ \gamma_{v}, & \mathrm{otherwise}. \end{array}\right. \end{array}$$

 □

**Proof of Theorem** **7.** We prove (63) from the right-hand side to the left.

(A29) $$\begin{array}{c} \prod_{v\in I_{\tau }}\alpha_{v|x}\prod_{v^{\prime }\in L_{\tau }}\left(1-\alpha_{v^{\prime }|x}\right)=\prod_{v\in I_{\tau }}\alpha_{v|x}\prod_{v^{\prime }\in L_{\tau }\cap L_{p}}\left(1-\alpha_{v^{\prime }|x}\right)\prod_{v^{\prime \prime }\in L_{\tau }\cap I_{p}}\left(1-\alpha_{v^{\prime \prime }|x}\right). \end{array}$$

In the following, we transform each of the above products in order. First, the first product is transformed by substituting (62) as follows.

(A30) $$\begin{array}{c} \prod_{v\in I_{\tau }}\alpha_{v|x}=\prod_{v\in I_{\tau }}\frac{\alpha_{v}g\left(x,v\right)\prod_{v^{\prime }\in {\mathrm{Ch}}_{p}\left(v\right)}q\left(x|v^{\prime }\right)}{q(x|v)}. \end{array}$$

Next, the second product is transformed as follows.

(A31) $$\begin{array}{cc} \prod_{v\in L_{\tau }\cap L_{p}}\left(1-\alpha_{v|x}\right) & =\prod_{v\in L_{\tau }\cap L_{p}}\left(1-\alpha_{v}\right) \end{array}$$

(A32) $$\begin{array}{cc} \; & =\prod_{v\in L_{\tau }\cap L_{p}}\frac{\left(1-\alpha_{v}\right)h\left(x,v\right)}{q(x|v)}, \end{array}$$

where (A31) is because of (62) and (A32) is because q(x|v)=h(x,v) for $v\in L_{p}$. Lastly, the third product is transformed as follows.

(A33) $$\begin{array}{cc} \prod_{v\in L_{\tau }\cap I_{p}}\left(1-\alpha_{v|x}\right) & =\prod_{v\in L_{\tau }\cap I_{p}}\left(1-\frac{\alpha_{v}g\left(x,v\right)\prod_{v^{\prime }\in {\mathrm{Ch}}_{p}\left(v\right)}q\left(x|v^{\prime }\right)}{q(x|v)}\right) \end{array}$$

(A34) $$\begin{array}{cc} \; & =\prod_{v\in L_{\tau }\cap I_{p}}\frac{q\left(x|v\right)-\alpha_{v}g\left(x,v\right)\prod_{v^{\prime }\in {\mathrm{Ch}}_{p}\left(v\right)}q\left(x|v^{\prime }\right)}{q(x|v)} \end{array}$$

(A35) $$\begin{array}{cc} \; & =\prod_{v\in L_{\tau }\cap I_{p}}\frac{\left(1-\alpha_{v}\right)h\left(x,v\right)}{q(x|v)}, \end{array}$$

where (A33) is because of (62) and (A35) is because of substitution of (61) into q(x|v) at the numerator. Therefore, we can combine (A30), (A32), and (A35). Then,

(A36) $$\begin{array}{cc} \left(A29\right) & =\prod_{v\in I_{\tau }}\frac{\alpha_{v}g\left(x,v\right)\prod_{v^{\prime }\in {\mathrm{Ch}}_{p}\left(v\right)}q\left(x|v^{\prime }\right)}{q(x|v)}\prod_{v^{\prime }\in L_{\tau }}\frac{\left(1-\alpha_{v^{\prime }}\right)h\left(x,v^{\prime }\right)}{q(x|v^{\prime })}. \end{array}$$

Here, (A36) is a telescoping product; i.e., q(x|v) appears at once each in the denominator and the numerator. Therefore, we can cancel them except for $q(x|v_{\lambda })$. Then,

(A37) $$\begin{array}{cc} \left(A36\right) & =\frac{1}{q(x|v_{\lambda })}\prod_{v\in I_{\tau }}\alpha_{v}g\left(x,v\right)\prod_{v^{\prime }\in L_{\tau }}\left(1-\alpha_{v^{\prime }}\right)h\left(x,v^{\prime }\right) \end{array}$$

(A38) $$\begin{array}{cc} \; & =\frac{1}{q(x|v_{\lambda })}\left(\prod_{v\in I_{\tau }}g\left(x,v\right)\prod_{v^{\prime }\in L_{\tau }}h\left(x,v^{\prime }\right)\right)\left(\prod_{v\in I_{\tau }}\alpha_{v}\prod_{v^{\prime }\in L_{\tau }}\left(1-\alpha_{v^{\prime }}\right)\right) \end{array}$$

(A39) $$\begin{array}{cc} \; & =\frac{p(x|\tau )p(\tau )}{q(x|v_{\lambda })}, \end{array}$$

where we used (58) and Definition 1. In addition, because of Theorem 4,

(A40) $$\begin{array}{c} q\left(x|v_{\lambda }\right)=E\left[p\left(x|T\right)\right]=\sum_{\tau \in T}p\left(x|\tau \right)p\left(\tau \right)=p\left(x\right). \end{array}$$

Therefore,

(A41) $$\begin{array}{c} \left(A39\right)=\frac{p(x|\tau )p(\tau )}{p(x)}=p\left(\tau |x\right). \end{array}$$

Then, Theorem 7 holds. □

**Proof of Corollary** **2.** We will prove only q(x|v)=1 for $v⋡v_{\mathrm{end}}$. Then, (66) and (67) are straightforwardly derived by substituting it with (64) and (65) into (61) and (62). For $v⋡v_{\mathrm{end}}$, by substituting (64) and (65) into (61),

(A42) $$\begin{array}{c} q\left(x|v\right)=\left\{\begin{array}{cc} 1, & v\in L_{p},\\ \left(1-\alpha_{v}\right)+\alpha_{v}\prod_{v^{\prime }\in {\mathrm{Ch}}_{p}\left(v\right)}q\left(x|v\right), & v\in I_{p}. \end{array}\right. \end{array}$$

Since this has the same form as (A6), q(x|v)=1 for $v⋡v_{\mathrm{end}}$ is derived in a similar manner. □

## Appendix B. Pseudocode to Calculate Mode

**Algorithm A1** Calculation of mode.
**Require:** ${\left\{\alpha_{v}\right\}}_{v\in V_{p}}$ **Ensure:** $\tau^{*}=\arg\max_{\tau }p\left(\tau \right)$ 1: **function**flag_calculation(*v*) ▹ Subroutine 2: **if** $v\in L_{p}$ **then** 3: $\delta_{v}\leftarrow 0$ 4: **return** 1 5: **else if** $v\in I_{p}$ **then** 6: $\mathrm{tmp}\leftarrow \prod_{v^{\prime }\in {\mathrm{Ch}}_{p}\left(v\right)}$flag_calculation($v^{\prime }$) 7: **if** $1-\alpha_{v}<\alpha_{v}\cdot \mathrm{tmp}$ **then** 8: $\delta_{v}\leftarrow 1$ 9: **return** $\alpha_{v}\cdot \mathrm{tmp}$ 10: **else** 11: $\delta_{v}\leftarrow 0$ 12: **return** $1-\alpha_{v}$ 13: **end if** 14: **end if** 15: **end function** 17: **function**backtracking(v,V,E) ▹ Subroutine 18: **if** $\delta_{v}=0$ **then** 19: **return** 20: **else if** $\delta_{v}=1$ **then** 21: $V\leftarrow V\cup {\mathrm{Ch}}_{p}\left(v\right)$ 22: $E\leftarrow E\cup {⋃}_{v^{\prime }\in {\mathrm{Ch}}_{p}\left(v\right)}\left(v,v^{\prime }\right)$ 23: **for all** $v^{\prime }\in {\mathrm{Ch}}_{p}\left(v\right)$ **do** 24: backtracking($v^{\prime },V,E$) 25: **end for** 26: **return** 27: **end if** 28: **end function** 30: **procedure** ▹ The main procedure 31: flag_calculation($v_{\lambda }$) 32: V←∅ 33: E←∅ 34: backtracking($v_{\lambda },V,E$) 35: **return** $\tau^{*}=\left(V,E\right)$ 36: **end procedure**
